# A rare cause of primary hypoparathyroidism due to a novel mutation in the GATA3 gene – the Barakat syndrome

**DOI:** 10.1186/1687-9856-2013-S1-P170

**Published:** 2013-10-03

**Authors:** SMY Wong, WM But, Angel Chan, W Chan

**Affiliations:** 1 Department of Paediatrics, Queen Elizabeth Hospital, Hong Kong; 2 Department of Pathology, Queen Elizabeth Hospital, Hong Kong

## 

Barakat syndrome, also known as HDR syndrome (hypoparathyroidism, deafness and renal dysplasia), is a rare autosomal dominant disorder, secondary to mutation of the GATA3 gene which is located at chromosome 10p. The GATA3 protein is one of the transcription factors which play an essential role in the embryonic development of the parathyroids, inner ears and kidneys.

We report a Chinese patient who presented with hypocalcaemic convulsion at day 11 of life due to primary hypoparathyroidism. Her serum calcium became normalized with calcium and vitamin D supplement. There was no other clinical feature to suggest DiGeorge syndrome and no family history of hypocalcaemia. The exact cause of hypoparathyroidism was not known at that time. At 6 months of age, she developed the first episode of E-coli urinary tract infection. Ultrasonogram of the kidney was normal but voiding cystogram revealed an intra-renal reflux of right kidney with dilated right ureter. Prophylactic antibiotics was prescribed. However, she developed repeated urinary tract infection with febrile seizures at 13 months and 15 months old respectively. Re-implantation of the right ureter was hence performed at 2 years of age. At the same time, she failed hearing screening test at her regular health assessment and audiometry showed bilateral hearing deficit. When she was reassessed at ten years of age, she enjoyed good health with only mildly deranged renal function.

In view of the presence of hypoparathyroidism, sensorineural hearing deficit and renal anomaly, Barakat syndrome was suspected and genetic analysis of the *GATA3* gene was performed at nine years of age. A heterozygous novel deletion mutation (c.925-3_925-2delCA) was detected in intron 3 of the *GATA3* gene. This mutation was not detected in her parents, suggesting that it is a *de novo* mutation. In conclusion, this is the first case report of a southern Chinese patient with Barakat syndrome diagnosed nine years after the initial presentation with hypoparathyroidism. Barakat syndrome is an extremely rare clinical entity and delayed diagnosis is not uncommon. Physicians should be aware of this condition for early diagnosis and family screening.

